# Preliminary Application of a Quantitative Collateral Assessment Method in Acute Ischemic Stroke Patients With Endovascular Treatments: A Single-Center Study

**DOI:** 10.3389/fneur.2021.714313

**Published:** 2021-12-23

**Authors:** Ruoyao Cao, Peng Qi, Yun Jiang, Shen Hu, Gengfan Ye, Yaxin Zhu, Ling Li, Zilong You, Juan Chen

**Affiliations:** ^1^Graduate School of Peking Union Medical College, Beijing, China; ^2^Department of Radiology, Beijing Hospital, National Center of Gerontology, Institute of Geriatric Medicine, Chinese Academy of Medical Sciences, Beijing, China; ^3^Department of Neurosurgery, Beijing Hospital, National Center of Gerontology, Institute of Geriatric Medicine, Chinese Academy of Medical Sciences, Beijing, China; ^4^Department of Neurology, Beijing Hospital, National Center of Gerontology, Institute of Geriatric Medicine, Chinese Academy of Medical Sciences, Beijing, China; ^5^Department of Neurosurgery, Ningbo Medical Center Lihuili Hospital, Ningbo, China; ^6^CT Clinical Research Department, CT Business Unit, Canon Medical Systems (China) Co., Ltd., Beijing, China; ^7^Beijing Institute of Geriatrics, Beijing Hospital, National Center of Gerontology, Institute of Geriatric Medicine, Chinese Academy of Medical Sciences, Beijing, China

**Keywords:** brain ischemia, collateral circulation, computed tomography angiography, CT perfusion, stroke

## Abstract

**Objectives:** To develop an efficient and quantitative assessment of collateral circulation on time maximum intensity projection CT angiography (tMIP CTA) in patients with acute ischemic stroke (AIS).

**Methods:** Eighty-one AIS patients who underwent one-stop CTA-CT perfusion (CTP) from February 2016 to October 2020 were retrospectively reviewed. Single-phase CTA (sCTA) and tMIP CTA were developed from CTP data. Ischemic core (IC) volume, ischemic penumbra volume, and mismatch ratio were calculated. The Tan scale was used for the qualitative evaluation of collateral based on sCTA and tMIP CTA. Quantitative collateral circulation (CCq) parameters were calculated semi-automatically with software by the ratio of the vascular volume (V) on both hemispheres, including tMIP CTA V_CCq_ and sCTA V_CCq._ Spearman correlation analysis was used to analyze the correlation of collateral-related parameters with final infarct volume (FIV). ROC and multivariable regression analysis were calculated to compare the significance of the above parameters in clinical outcome evaluation. The analysis time of the observers was also compared.

**Results:** tMIP CTA V_CCq_ (*r* = 0.61, *p* < 0.01), IC volume (*r* = 0.66, *p* < 0.01), Tan score on tMIP CTA (*r* = 0.52, *p* < 0.01) and mismatch ratio (*r* = 0.60, *p* < 0.01) showed moderate negative correlations with FIV. tMIP CTA V_CCq_ showed the best prognostic value for clinical outcome (AUC = 0.93, *p* < 0.001), and was an independent predictive factor of clinical outcome (OR = 0.14, *p* = 0.009). There was no difference in analysis time of tMIP CTA V_CCq_ among observers (*p* = 0.079).

**Conclusion:** The quantitative evaluation of collateral circulation on tMIP CTA is associated with clinical outcomes in AIS patients with endovascular treatments.

## Introduction

Reperfusion therapies significantly improve the prognosis of patients with acute ischemic stroke (AIS). With the popularization of endovascular treatments (EVTs) in AIS, it is necessary to build an individualized evaluation system, helping physicians make clinical decisions and predict outcomes before invasive intervention (1, 2). Previous studies have indicated that collateral circulation is one of the main factors determining ischemic penumbra (3–6). Comprehensive and accurate evaluation of collateral circulation is a necessary complement to develop individualized treatments for AIS patients.

The visibility of the collateral circulation on computed tomography angiography (CTA) strongly depends on the acquisition time. The strength of the collateral flow is more important to tissue fate than the velocity of collateral filling (7). Because the interindividual collateral circulation (distribution, filling time, etc.) is highly variable and the optimal acquisition time is individually different, four-dimensional CTA (4D CTA) obtained from the perfusion data (multi-time frame) was applied to evaluate collateral status more accurately (7–9). Time maximum intensity projection angiography (tMIP CTA), also known as timing-invariant (TI) CTA (10) or temporally fused maximum intensity projection (tMIP) CTA (7), reflects the maximum value on all projection planes scrolling over time, producing a new volume-based data packet that is generated from all phases images of CT perfusion (CTP). tMIP CTA may eliminate the drawback of single-phase CTA (sCTA) wherein collateral vessels are usually displayed incompletely due to delayed pathophysiology status, building a system with high temporal and spatial resolution (7, 8, 10–12). Thus, tMIP CTA improves the signal-to-noise ratio (SNR) and contrast-to-noise ratio (CNR) of images and may therefore be an ideal option for assessing collateral circulation (7, 8, 12).

Until now, there has been no unified collateral circulation evaluation system. Several grading scales have been applied in collateral circulation evaluation, including the CTA-based Miteff scale (13), the modified Tan scale (14), and the regional leptomeningeal collateral score (rLMC) (15). It is difficult to verify the predictive value, reliability, and validity of these different scoring systems. Therefore, a standardized evaluation model is needed. Here, we adopted a simple and efficient quantitative assessment based on tMIP CTA to evaluate collateral circulation status.

In this study, we assessed whether this new quantitative assessment system was able to evaluate collateral circulation accurately in comparison with the qualitative collateral score system and CTP. Additionally, the analysis time among observers with different levels of experience was also compared. We aimed to identify an efficient and highly accurate quantitative method for evaluating collateral circulation during the decision-making process.

## Methods

This study was approved by our institutional review board. Because patient information was analyzed retrospectively and anonymously, informed consent was waived.

### Study Population

The clinical data and imaging data of AIS patients with occlusion of the anterior circulation vessels who had received EVTs from February 2016 to October 2020 were retrospectively analyzed. Inclusion criteria: (1) acute stroke (onset time <24 h); (2) one-stop CT scan indicating occlusion of unilateral anterior circulation vessels [including the internal carotid artery (ICA) and/or the M1 segment of the middle cerebral artery (MCA)]; and (3) integral clinical and imaging data. Exclusion criteria: (1) intracranial hemorrhage; (2) old infarct size ≥ 2/3 of the MCA territory; (3) moderate to severe stenosis of ipsilateral proximal or contralateral arteries; (4) severe dysfunction of the heart, liver, lungs, and kidneys as well as hematological diseases; and (5) history of iodine allergy (16).

The AIS patients' clinical and imaging data were obtained from the medical records, including the National Institutes of Health Stroke Scale (NIHSS) score, Modified Rankin Scale (mRS) score, Alberta Stroke Program Early CT Score (ASPECTS), and related risk factors. The mRS score was used to evaluate the patients' functional outcomes. mRS scores of 0-2 indicate a good outcome, while mRS scores of 3-6 indicate a poor outcome. If patients died during follow-up, the score was recorded as 6. Recanalization was defined as a modified Thrombolysis in Cerebral Infarction grade 2b or 3 (17). The area of low-density on follow-up non-contrast CT (NCCT) images or high signal intensity on MR T2WI or DWI after 3-7 days was considered as the final infarct volume (FIV) (18).

### Scanning Protocol

All patients underwent a one-stop CTA-CTP scan on a 320 × 0.5 mm detector row CT scanner (Aquilion ONE, Canon Medical Systems) on admission according to our emergency procedures. An NCCT scan was performed first with the following scan parameters: 120 kV/200 mAs/detector and volume scanning, followed by one-stop dynamic volume 4D CTA-CTP scanning, reconstruction with adaptive iterative dose reduction, and layer thickness 0.5 mm. The contrast agent was injected through the elbow vein with an 18G intravenous catheter, while 40-50 ml of non-ionic contrast agent and 30 ml of saline were sequentially injected through a double-channel high-pressure injector according to the patient's weight (0.6 ml/kg). A CTP scan was performed 7 s after contrast injection with the following scanning parameters: 80 kV, 100 mAs, cover range of 16 cm, reconstruction with adaptive iterative dose reduction, and layer thickness 0.5 mm.

### Image Postprocessing

A total of 6,080-frame dynamic volume data in 19 phases of the whole brain were obtained. (1) sCTA: The arterial phase images (the phase with the maximum CT value of the artery) were selected from 19 phases as sCTA images. (2) tMIP CTA: tMIP CTA images were generated from all 19 phases using the analysis software installed in the CT workstation (Aquilion ONE, Canon Medical Systems). tMIP images reflect the maximum value of each matrix in the dynamic data for all phases (12). (3) CTP: Vitrea (Vital Images, Minnetonka, Minnesota) was used to perform all the CTP analyses. The input artery and output vein were obtained from the intracranial segment of the internal carotid artery in the normal side and superior sagittal sinus, respectively, and time-density curves (TDC) were obtained. A 38% reduction in cerebral blood volume (CBV) with a 5.3-s increase in time to peak (TTP) indicated infarct core (IC), and a 5.3-s increase in TTP without CBV reduction indicated ischemic penumbra (IP). The mismatch ratio was the IP volume divided by the IC volume (19).

Colt burden score (CBS) was defined as the extent of thromboembolic vessels of the anterior circulation based on CTA. Occlusions of the supraclinoid ICA, proximal and distal M1 segment were counted as two points, respectively, while each segment of M2 branches, anterior cerebral artery, and infraclinoid ICA occlusion were counted as one point, respectively. If thromboembolism was seen in any segment, the corresponding point was subtracted from 10. The remaining points constituted the final score (20).

### Evaluation Methods for the Collateral Status Based on sCTA and tMIP CTA

#### Quantitative Method

**Figure 1:**
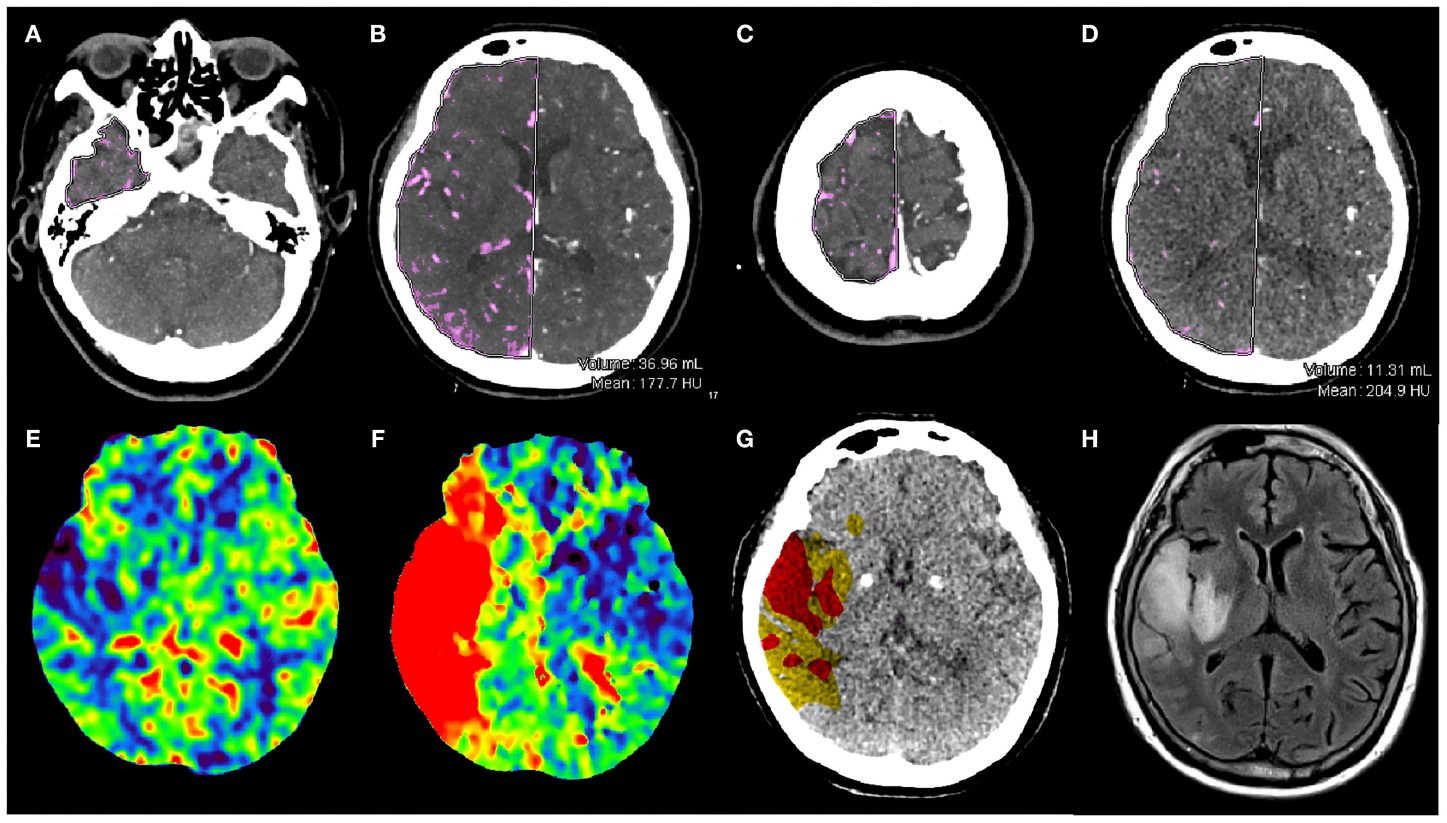
Illustrative example. Baseline one-stop CTA-CTP images and follow-up MRI in a 78-year-old woman with sudden weakness of the left upper limb. The baseline NIHSS score was 5. First, the layers were drawn manually in the right hemisphere on tMIP CTA: the temporal pole layer **(A)**, the basal ganglia layer **(B)** and the parietal lobe layer **(C)** were selected as the initial layer, the intermediate layer and the end layer, respectively. The blood vessel volume calculated automatically by the software, which was 36.96 ml in the right hemisphere. Then, the same methods were used to measure the vascular volume in the left hemisphere (normal side). tMIP CTA V_CCq_ was calculated by the ratio the volume of the affected hemisphere to that of the unaffected hemisphere. The **(D)** image shows the basal ganglia layer on sCTA, and the same method was used to calculate the volume of blood vessels in the right hemisphere as 11.31 ml. And the ratio the volume of the affected hemisphere to that of the unaffected hemisphere was sCTA V_CCq_. CBV at the affected hemisphere was decreased in the infarction area **(E)**, TTP was obviously prolonged **(F)**. Tissue map shows an IC area (red) of 50.23 ml and IP area (yellow) of 88.78 ml **(G)**. This patient underwent endovascular treatments and had a FIV of 32.86 mL at T2 Flair **(H)**. The 90-day mRS score for this patient was 0.

Volume Calc semi-automated volumetric measurement analysis software (Aquilion ONE; Canon Medical Systems Corporation) was used for the quantitative evaluation of collateral circulation. The detailed calculation process of this software was as follows. Step 1: Set thresholds of CT values including the minimum and maximum CT values in the region of interest (ROI) for calculating blood vessel volumes. In this study, the CT values of the intracranial arteries were measured on axial images (0.5-mm layer thickness). The minimum CT value was set as 100 HU (≥CT value of brain tissue and muscle after enhanced scan), and the maximum CT value was defined as the CT value of segment C1 of the internal carotid artery. Step 2: The temporal pole layer, the basal ganglia layer, and the parietal lobe layer were selected as the initial layer, the intermediate layer, and the end layer, respectively. The volume of the blood vessels was calculated automatically by the software. The skull and cerebellar hemispheres were avoided as much as possible when drawing the ROIs (Figure 1). The quantitative measurement of the collateral circulation (CCq) was expressed as a ratio and defined by the following formulas:


$$\begin{array}{l} V_{\text{CCq}}=V_{\text{affected}}/V_{\text{unaffected}},V_{\text{CCq}}\geq \;0; \end{array}$$

The volume (V) of the collateral vessels on the affected and unaffected sides were calculated automatically on tMIP CTA and sCTA, respectively, yielding the tMIP CTA vascular volume ratio (tMIP CTA V_CCq_) and the sCTA vascular volume ratio (sCTA V_CCq_).

#### Qualitative Method

Collateral vessels were assessed on tMIP CTA (Tan score on tMIP CTA) and sCTA (Tan score on sCTA) by using a four-point Modified Tan grading system. Collaterals were classified as good (2-3 scores) if there were 50% flow or more of the normal side revealed, and as poor (0-1 score) if <50% flow of the normal side was revealed.

### Analysis Time

In this study, image postprocessing and diagnosis were performed by three observers. Observer A was a junior radiologist with 2 years of work experience but they had never performed CT image postprocessing. Observer B was an intermediate radiologist who performed imaging diagnoses for approximately 5 years. Observer C was a senior radiologist who performed imaging diagnosis for over 15 years and was well-experienced in CT image postprocessing. The three observers received 20-30 min of basic training about CTP and tMIP image postprocessing.

The analysis time of quantitative collateral assessment on tMIP CTA included the time generating the tMIP CTA images, the time drawing the ROIs, and the time required by the software to calculate the vascular volume automatically. The time spent on quantitative collateral assessment on sCTA was also recorded, including the time selecting the appropriate arterial phase from the 19 phases, the time drawing the ROIs on all three layers, and the time required by the software to calculate the vascular volume automatically. Meanwhile, the analysis time also included the time of selecting the appropriate arterial phase, time generating the tMIP CTA images, and time of evaluation.

The analysis time of CTP included the time of TDC adjustment and the Vitrea software automatic postprocessing.

### Statistical Analysis

Normally distributed quantitative data were expressed as the means ± standard errors of the mean (SEMs); non-normally distributed quantitative data were expressed as the medians (Q1, Q3); qualitative data were expressed as the frequencies (percentages). Both parametric and non-parametric statistical tests were used, as the data were skewed. The chi-squared test was performed to test qualitative data. The non-parametric Mann-Whitney *U*-test was used to test non-normally distributed quantitative data. The unpaired Student's *t*-test was used to test normally distributed quantitative data. Pairwise comparisons were performed using Fisher's least significant difference (LSD). All patients were assessed twice by each observer independently. Interobserver agreement was described by the intraclass correlation coefficient (ICC), and *p* < 0.05 was considered statistically significant. Spearman correlation analysis was used to analyze the correlation of collateral-related parameters with FIV. Logistic regression was used to analyze the risk factors of clinical outcomes. The variables in the regression were selected by the Akaike information criterion (AIC). Receiver operating characteristic (ROC) curves were used to evaluate the predictive ability of the above parameters, and the sensitivity, specificity, area under the curve (AUC), and 95% confidence interval (95% CI) were calculated. The results were considered significant for two-tailed *p* < 0.05. Statistical analysis was performed using Statistical Package for the Social Sciences ver. 25 software (SPSS, Chicago, IL).

## Results

### Study Population

In total, 81 patients were enrolled in this study, including 41 males (50.6%) and 40 females (49.4%). Compared with those in the poor outcome group, patients with good outcomes showed lower baseline NIHSS scores, younger ages, lower incidences of atrial fibrillation, lower incidences of a previous stroke and/or coronary heart disease, better collateral vessels, less IC volume, and higher mismatch ratio (all, *p* < 0.05). Detailed information was summarized in Table 1.

**Table 1:** Characteristics of patients with good and poor outcome.

**Variables**	**Poor outcome ***n*** = 36**	**Good outcome ***n*** = 45**	***U/χ2***	***P***
Age, median [IQR]	81.00(72.25,84.00)	71.00(61.50,81.50)	499.50	0.003
Male, *n* (%)	15(41.70)	26(57.80)	2.08	0.150
Baseline NIHSS score, median [IQR]	15.50(11.25,21.00)	10.00(6.00,15.00)	421.00	<0.001
IV-tPA, *n* (%)	8(22.20)	14(31.10)	0.80	0.371
Atrial fibrillation, *n* (%)	23(63.90)	17(37.80)	5.46	0.020
Hypertension, *n* (%)	31(86.10)	33(73.30)	1.97	0.161
Diabetes mellitus, *n* (%)	17(47.20)	12(26.70)	3.68	0.055
Dyslipidemia, *n* (%)	16(44.40)	15(33.30)	1.05	0.307
Smoking, *n* (%)	10(27.80)	16(35.60)	0.56	0.456
Previous stroke, *n* (%)	15(41.70)	9(20.00)	4.50	0.034
Coronary disease, *n* (%)	19(52.80)	14(31.10)	3.89	0.049
Anticoagulant, *n* (%)	3(8.30)	5(11.10)	0.17	0.677
ASPECTS,median [IQR]	6.50(3.00,8.00)	8.00(6.00,8.00)	636.00	0.094
Clot burden score, median [IQR]	4.00(0.00,7.00)	6.00(2.00,9.00)	626.50	0.076
Tan score on sCTA, median [IQR]	0.00(0.00,1.75)	1.00(0.00,2.00)	681.00	0.187
Tan score on tMIP CTA, median [IQR]	1.00(1.00,2.00)	3.00(2.00,3.00)	322.00	<0.001
Recanalization, *n* (%)	28(77.80)	41(91.10)	2.82	0.053
FIV (mL), median [IQR]	98.97(44.37,223.85)	16.25(4.79,46.63)	275.00	<0.001
tMIP CTA V_CCq_, median [IQR]	0.53(0.34,0.71)	0.97(0.91,1.06)	112.00	<0.001
sCTA V_CCq_, median [IQR]	0.44(0.33,0.55)	0.63(0.48,0.81)	389.00	<0.001
IC volume (ml), median [IQR]	89.41(52.00,134.06)	90.06(49.07,140.51)	713.00	0.724
IP volume (ml), median [IQR]	26.99(10.41,47.56)	72.58(13.95,145.23)	440.00	0.002
Mismatch ratio, median [IQR]	1.83(0.66,4.44)	3.65(2.01,6.67)	450.50	0.003

*IQR, interquartile range; NIHSS, National Institutes of Health Stroke Scale; ASPECTS, Alberta Stroke Program Early CT Score; SD, standard deviation; IV-tPA, intravenous recombinant tissue-type plasminogen activator; FIV, final infarct volume; tMIP CTA, time maximum intensity projection computed tomography angiography; sCTA, single-phase computed tomography angiography; tMIP CTA V_CCq_, quantitative measurement of vascular volume ratio on tMIP CTA; sCTA V_CCq_, quantitative measurement of vascular volume ratio on sCTA; IC, ischemic core; IP, ischemic penumbra*.

### Relationship of Collateral Circulation With FIV

FIV was assessed on NCCT (14 patients) or MRI (67 patients). tMIP CTA V_CCq_ (*r* = 0.61, *p* < 0.01), IC volume (*r* = 0.66, *p* < 0.01), Tan score on tMIP CTA (*r* = 0.52, *p* < 0.01) and Mismatch ratio (*r* = 0.60, *p* < 0.01) showed moderate negative correlations with FIV. Patients were further divided into two groups: recanalization group (*n* = 69) and non-recanalization group (*n* = 12). In the group of non-recanalization, all of tMIP CTA V_CCq_ (*r* = 0.63, *p* < 0.05), sCTA V_CCq_ (*r* = 0.53, *p* < 0.05), Tan score on tMIP CTA (*r* = 0.56, *p* < 0.05) and IC volume (*r* = 0.65, *p* < 0.05) had moderate negative correlations with FIV (Table 2).

**Table 2:** Association of collateral-related parameters with FIV in AIS patients after EVTs.

**Variables**	**Total** **(***n*** = 81)**	**Recanalization (***n*** = 69)**	**Non-recanalization (***n*** = 12)**
tMIP CTA V_CCq_	−0.61**	−0.58**	−0.63*
sCTA V_CCq_	−0.38*	−0.26*	−0.53*
Tan score on tMIP CTA	−0.52**	−0.50**	−0.56*
Tan score on sCTA	−0.23*	−0.31**	−0.26
IP volume	0.17	0.17	0.21
IC volume	0.66**	0.66**	0.65*
Mismatch ratio	−0.60**	−0.63**	−0.34

*FIV, final infarct volume; tMIP CTA, time maximum intensity projection computed tomography angiography; sCTA, single-phase computed tomography angiography; tMIP CTA V_CCq_, quantitative measurement of vascular volume ratio on tMIP CTA; sCTA V_CCq_, quantitative measurement of vascular volume ratio on sCTA; IP, ischemic penumbra; IC, ischemic core*.

*All correlation coefficients are presented as Spearman r values*.

* *P < 0.05*.

** *P < 0.01*.

### Association of Collateral Circulation and Clinical Outcome

In multivariate logistic regression analysis, tMIP CTA V_CCq_ (odds ratio = 0.14; 95%CI, 0.03-0.59; *p* = 0.009) was an independent predictive factor of outcome (Table 3). The area under the curve (AUC) of indicators for predicting clinical outcome was obtained by receiver operating characteristic (ROC) curve analysis. The results are shown in Table 4: tMIP CTA V_CCq_ had the best value in predicting clinical outcome (AUC, 0.93; sensitivity, 0.88; specificity, 0.80, *p* < 0.001).

**Table 3:** Association between collateral-related parameters and clinical outcome.

**Variables**	**β**	**SE**	**Wald χ2**	**Exp(B)**	**Lower 95% CI**	**Upper 95% CI**	***P***
tMIP CTA V_CCq_	−1.96	0.73	6.78	0.14	0.03	0.59	0.009
sCTA V_CCq_	0.20	4.22	0.00	1.22	0.00	4759.09	0.962
Tan score on tMIP CTA	−1.39	1.01	1.89	0.25	0.04	1.81	0.169
Tan score on sCTA	1.66	0.82	0.93	5.25	1.06	26.12	0.336
IP volume	−0.01	0.01	1.07	0.99	0.97	1.01	0.300
IC volume	0.01	0.03	0.07	1.01	0.96	1.06	0.798
Mismatch ratio	0.08	0.15	0.28	1.08	0.81	1.46	0.594

*FIV, final infarct volume; CI, confidence interval; tMIP CTA, time maximum intensity projection computed tomography angiography; sCTA, single-phase computed tomography angiography; tMIP CTA V_CCq_, quantitative measurement of vascular volume ratio on tMIP CTA; sCTA V_CCq_, quantitative measurement of vascular volume ratio on sCTA; IP, ischemic penumbra; IC, ischemic core*.

**Table 4:** Receiver operating characteristics curves of collateral-related parameters for evaluating clinical outcome.

**Variables**	**AUC (95% CI)**	**Sensitivity (95% CI)**	**Specificity (95% CI)**	**Youden index**	**Associated criterion**	***P***
tMIP CTA V_CCq_	0.93(0.85,1.00)	0.88(0.56,0.94)	0.80(0.39,1.00)	0.68	−0.84	<0.001
sCTA V_CCq_	0.74(0.63,0.85)	0.71(0.38,0.94)	0.66(0.39,0.86)	0.47	0.03	<0.001
Tan score on sCTA	0.56(0.43,0.70)	0.74(0.28,0.99)	0.73(0.19,0.99)	0.47	2.50	0.307
Tan score on tMIP CTA	0.79(0.68,0.89)	0.50(0.07,0.90)	0.98(0.30,1.00)	0.48	−1.00	<0.001
IP volume	0.52(0.39,0.65)	0.50(0.23,0.79)	0.49(0.27,0.75)	0.00	248.96	0.727
IC volume	0.71(0.58,0.83)	0.65(0.47,0.77)	0.60(0.25,0.94)	0.25	52.69	0.002
Mismatch ratio	0.70(0.57,0.82)	0.71(0.44,0.82)	0.61(0.26,0.90)	0.32	9.05	0.002

*AUC, area under the curve; CI, confidence interval; tMIP CTA, time maximum intensity projection computed tomography angiography; sCTA, single-phase computed tomography angiography; tMIP CTA V_CCq_, quantitative measurement of vascular volume ratio on tMIP CTA; sCTA V_CCq_, quantitative measurement of vascular volume ratio on sCTA*.

### Analysis Time

For tMIP CTA V_CCq_, observer A, B, and C had a statistically comparable analysis time (110.43 ± 5.44 s vs. 106.86 ± 6.27 s vs. 101.71 ± 8.31 s, *p* = 0.079). For CTP, sCTA V_CCq_, Tan score on tMIP CTA, and Tan score on sCTA, there were significant differences among three observers in analysis time (all, *p* < 0.001) (Table 5).

**Table 5:** Comparison of analysis time of doctors of different grades.

**Variables**	**Junior doctor**	**Intermediate doctor**	**Senior doctor**	***F***	***P***
Tan score on sCTA (s), mean ± SD^ab^	94.43 ± 7.28	57.57 ± 7.89	20.43 ± 7.89	161.95	<0.001
Tan score on tMIP CTA (s), mean ± SD^ab^	75.43 ± 7.57	44.43 ± 8.06	20.00 ± 4.08	116.67	<0.001
sCTA V_CCq_ (s), mean ± SD^ab^	142.29 ± 14.04	120.86 ± 14.06	115.71 ± 7.27	20.28	<0.001
tMIP CTA V_CCq_ (s), mean ± SD	110.43 ± 5.44	106.86 ± 6.27	101.71 ± 8.31	2.93	0.079
CTP (s), mean ± SD^ab^	152.57 ± 12.22	130.43 ± 8.30	98.43 ± 8.54	53.44	<0.001

a *, The P-value of LSD in Junior Doctor vs. Intermediate Doctor < 0.05*.

b *, The P-value of LSD in Junior Doctor vs. Senior Doctor < 0.05*.

*LSD, least significant difference; tMIP CTA, time maximum intensity projection computed tomography angiography; sCTA, single-phase computed tomography angiography; tMIP CTA V_CCq_, quantitative measurement of vascular volume ratio on tMIP CTA; sCTA V_CCq_, quantitative measurement of vascular volume ratio on sCTA; CTP, computed tomography perfusion*.

The ICC among the three observers was 0.96 (tMIP CTA V_CCq_), 0.86 (sCTA V_CCq_), 0.86 (Tan score on tMIP CTA), 0.67 (Tan score on sCTA), and 0.97 (CTP).

## Discussion

Good collateral circulation contributes to maintaining the blood supply around an ischemic lesion for a relatively long time to preserve penumbra tissue (21, 22). Although evaluation of the collateral circulation has not yet been written into current guidelines of treatment for acute ischemic stroke, it is well-known that collateral circulation plays an important role in the development of cerebral infarction. Clinically, the collateral circulation influences the selection of treatment strategies and prognoses of AIS patients.

We found that tMIP CTA V_CCq_ (*r* = 0.61, *p* < 0.01), IC volume (*r* = 0.66, *p* < 0.01), Tan score on tMIP CTA (*r* = 0.52, *p* < 0.01) and the mismatch ratio (*r* = 0.60, *p* < 0.01) showed moderate negative correlations with FIV. In the group of non-recanalization, tMIP CTA V_CCq_ (*r* = 0.63, *p* < 0.05), sCTA V_CCq_ (*r* = 0.53, *p* < 0.05), Tan score on tMIP CTA (*r* = 0.56, *p* < 0.05) and IC volume (*r* = 0.65, *p* < 0.05) had moderate negative correlations with FIV. Moreover, the tMIP CTA VCCq was an independent predictor of good outcome in AIS patients (OR = 0.14; 95% CI: 0.03, 0.59; *p* = 0.009), and had the best value in predicting clinical outcome (AUC, 0.93; sensitivity, 0.88; specificity, 0.80, *p* < 0.001).

Although an increasing number of studies have focused on the role of collateral circulation in clinical treatment decisions, few studies have evaluated intracranial collateral vessels using quantitative analysis (23, 24). Most evaluation methods of the collateral circulation focus on rating scales, where doctors are required to be familiar with the rating rules, and the consistency among observers is low (13, 15). For clinicians, especially junior and intermediate doctors, a reliable quantitative method for evaluating collateral vessels could help them make individualized treatment options more accurately and effectively.

To date, only a few studies have adopted the quantitative evaluation of collateral vessels. Shi et al. reported that measuring the maximum blood flow of the collateral vessels in the Sylvian fissure on CT perfusion images was a feasible method for quantitatively evaluating the collateral circulation, which was related to clinical outcomes in AIS patients (23). Boers et al. proposed that automatic quantitative collateral circulation assessment in AIS patients based on sCTA could be helpful for developing treatment options (24). In our prior study, we reported that tMIP CTA, which integrates the information from multiple CTA phases and displays the maximum CT value of all projection planes, showed higher image quality of intracranial vascularity than sCTA did and then improved the accuracy of collateral circulation evaluation (12). In this study, we performed a further quantitative evaluation of the intracranial collateral circulation based on tMIP CTA.

Previous studies revealed that better collateral status indicates a smaller infarct volume, larger penumbra volume, and decreased infarct growth (21, 25, 26). The results of this study demonstrated that a higher volume ratio of the collateral vessels predicted a better outcome and smaller FIV, and tMIP CTA V_CCq_ had the best predictive value for prognosis. For patients who failed to get successful recanalization, collateral circulation was also an important determinant of FIV, which helped to maintain the blood supply around the FIV for a relatively long period after stroke onset and preserve salvable brain tissue (27). In addition, the above results indicate that sCTA, the most applied technique for AIS patients, may underestimate the status of the collateral circulation due to the lack of time information, whereas tMIP CTA provided more accurate results when evaluating the collateral circulation by integrating the vascular information from multiple phases, including the delayed development of collateral vessels. CTP is also an alternative method for evaluating collateral circulation, however, it is worth noting that CTP parameters may be influenced by variations in postprocessing and algorithms (28, 29). The tMIP CTA V_CCq_ contained complete blood vessel information and was the most accurate parameter for collateral circulation evaluation when compared with the other parameters. Furthermore, it was noticed that 20 patients had better collateral circulation before EVTs (tMIP CTA V_CCq_ > 1) and good clinical outcomes (mRS scores 0-2). Among them, 12 patients had an mRS score of 0 point, 6 patients had 1 point, and 2 patients had 2 points.

It should be mentioned that 40.6% (28/69) of our patients had poor outcomes after vascular recanalization (Table 1). This may be due to their old age and many complications, such as coronary heart disease and atrial fibrillation.

We also recorded the analysis time to investigate whether evaluations with different methods were correlated with diagnostic experience. In this study, the analysis time of Tan score on sCTA, Tan score on tMIP CTA, sCTA V_CCq_, and CTP were related to the diagnostic experience level, while the analysis time of tMIP CTA V_CCq_ did not. The reason might be that tMIP CTA is a new volume packet where software automatically integrates all phases of CTA information, and the selection time of the optimal arterial phase was not needed. Short-term training (~20-30 min) is enough for the inexperienced doctors to quantitatively evaluate collateral vessel status using the software, and it is possible to obtain reliable results with no increase in evaluating time of tMIP V_CCq_. Currently, the selection of AIS patients for reperfusion therapy based on a traditional time window is disadvantaged, while tissue window plays a more important role in clinical decision-making (30, 31). However, the tissue window of individual patients is not fixed, depending on several compensation mechanisms, including collateral compensation. Using appropriate imaging modalities before reperfusion therapy in suitable patients, especially mechanical thrombectomy, may benefit patients with AIS. At present, many grading scales are available for collateral status assessment in ischemic stroke (13–15). The rLMC score is more comprehensive when evaluating collateral circulation. However, the rLMC score requires evaluation of nine anatomical regions, which is relatively complicated and time-consuming (15, 32). The Miteff score mainly focuses on the surface of the leptomeninges, the sylvian fissure area, and the distal end of the vascular occlusion, so it may ignore other important functional areas for collateral blood flow compensation (13, 22). The Tan score evaluates the collateral circulation based on the range of collateral blood flow after vascular occlusion (14). Although the Tan score is slightly rough, a previous study has shown that the reliability of the Tan score is higher than the Miteff score (22). Meanwhile, the Tan score may be easier for the doctors to assess collateral status. Conversely, a study has shown that the collateral circulation defined by the Tan score is one of the risk factors for patients receiving acute endovascular treatment (21). In general, each of the above methods has respective advantages and disadvantages. In this study we focused on quantitative collateral circulation evaluation methods, so we chose the Tan score to represent the qualitative assessment of collateral circulation. Perfusion parameters were also added to verify the accuracy of the tMIP CTA V_CCq_.

The limitations of this study are as follows. First, it is a single-center retrospective study, and the sample size is relatively small. Second, the tMIP technique may enhance any artifacts present in the posterior cranial fossa, which is why patients with posterior circulation stroke were not included in this study. Third, tMIP technology includes arterial and venous information. Although most of the current studies focused on arterial collateral circulation studies, there were studies reporting that the venous system has a greater role in assessing ischemia than previously described (33). In the near future, a larger scale prospective trial is needed to further confirm our findings. We did not include multiphase CTA (mCTA) in this study because the evaluation time on mCTA is much more than that on tMIP CTA and sCTA.

## Conclusion

Quantitative measurement of tMIP CTA is a reliable and time-saving method for assessing collateral circulation. It may help clinicians develop more precise treatment strategies and can aid in predicting clinical outcomes. In addition, compared with sCTA V_CCq_, qualitative parameters, and CTP parameters, quantitative measurement on tMIP CTA had the best predictive value for patient outcome. We hope this accurate and effective evaluation of the collateral circulation will provide clinicians with one more option that helps them to choose an appropriate treatment strategy.

## Data Availability Statement

The raw data supporting the conclusions of this article will be made available by the authors, without undue reservation.

## Ethics Statement

The studies involving human participants were reviewed and approved by Beijing Hospital institutional review board. The patients/participants provided their written informed consent to participate in this study. Written informed consent was obtained from the individual(s) for the publication of any potentially identifiable images or data included in this article.

## Author Contributions

JC conceived and designed the study. PQ, YJ, GY, LL, and ZY collected the data. PQ, YJ, and YZ were responsible for quality control. RC and JC conceived of the project, analyzed the data, and wrote the paper. All authors helped, organized, carried out the research, read, and approved the final manuscript.

## Funding

The authors declare that this study received funding from 2020 SKY Imaging Research Fund of the Chinese International Medical Foundation (project No. Z-2014-07-2003-02), 2020 Beijing Hospital National Natural Science Foundation of China Preliminary Research Project (project No. BJ-2020-131).

## Conflict of Interest

YZ is employed by Canon Medical System (China) Co., Ltd. The remaining authors declare that the research was conducted in the absence of any commercial or financial relationships that could be construed as a potential conflict of interest.

## Publisher's Note

All claims expressed in this article are solely those of the authors and do not necessarily represent those of their affiliated organizations, or those of the publisher, the editors and the reviewers. Any product that may be evaluated in this article, or claim that may be made by its manufacturer, is not guaranteed or endorsed by the publisher.

## References

[1] Albers GW Marks MP Kemp S Christensen S Tsai JP Ortega-Gutierrez S. Thrombectomy for stroke at 6 to 16 hours with selection by perfusion imaging. N Engl J Med. (2018) 378:708–18. 10.1056/NEJMoa1713973 29364767 PMC6590673

[2] Menon BK D'Esterre CD Qazi EM Almekhlafi M Hahn L Demchuk AM. Multiphase CT angiography: a new tool for the imaging triage of patients with acute ischemic stroke. Radiology. (2015) 275:510–20. 10.1148/radiol.15142256 25633505

[3] Galego O Jesus-Ribeiro J Baptista M Sargento-Freitas J Martins AI Silva F. Collateral pial circulation relates to the degree of brain edema on CT 24 hours after ischemic stroke. Neuroradiol J. (2018) 2018:1092990591. 10.1177/1971400918769912 29663853 PMC6136138

[4] Kim BM Baek J Heo JH Nam HS Kim YD Yoo J. Collateral status affects the onset-to-reperfusion time window for good outcome. J Neurol Neurosurg Psychiatry. (2018) 89:903–9. 10.1136/jnnp-2017-317627 29519900

[5] Boers AM Jansen IG Berkhemer OA Yoo AJ Lingsma HF Slump CH. Collateral status and tissue outcome after intra-arterial therapy for patients with acute ischemic stroke. J Cerebral Blood Flow Metab. (2016) 37:3589–98. 10.1177/0271678X16678874 27864462 PMC5669341

[6] Flores A Rubiera M Ribó M Pagola J Rodriguez-Luna D Muchada M. Poor collateral circulation assessed by multiphase computed tomographic angiography predicts malignant middle cerebral artery evolution after reperfusion therapies. Stroke. (2015) 46:3149–53. 10.1161/STROKEAHA.115.010608 26419969

[7] Frölich AMJ Wolff SL Psychogios MN Klotz E Schramm R Wasser K. Time-resolved assessment of collateral flow using 4D CT angiography in large-vessel occlusion stroke. Eur Radiol. (2014) 24:390–6. 10.1007/s00330-013-3024-6 24078013

[8] Smit EJ Vonken EJ van Seeters T Dankbaar JW van der Schaaf IC Kappelle LJ. Timing-Invariant imaging of collateral vessels in acute ischemic stroke. Stroke. (2013) 44:2194–9. 10.1161/STROKEAHA.111.000675 23760216

[9] Vilela P Rowley HA. Brain ischemia: CT and MRI techniques in acute ischemic stroke. Eur J Radiol. (2017) 96:162–72. 10.1016/j.ejrad.2017.08.014 29054448

[10] Smit EJ Vonken EJ van der Schaaf IC Mendrik AM Dankbaar JW Horsch AD. Timing-invariant reconstruction for deriving high-quality CT angiographic data from cerebral CT perfusion data. Radiology. (2012) 263:216–25. 10.1148/radiol.11111068 22332063

[11] Murayama K Suzuki S Matsukiyo R Takenaka A Hayakawa M Tsutsumi T. Preliminary study of time maximum intensity projection computed tomography imaging for the detection of early ischemic change in patient with acute ischemic stroke. Medicine. (2018) 97:e9906. 10.1097/MD.0000000000009906 29489691 PMC5851726

[12] Cao R Jiang Y Lu J Wu G Zhang L Chen J. Evaluation of intracranial vascular status in patients with acute ischemic stroke by time maximum intensity projection CT angiography: a preliminary study. Acad Radiol. (2020) 27:696–703. 10.1016/j.acra.2019.06.013 31324580

[13] Miteff F Levi CR Bateman GA Spratt N Mcelduff P Parsons MW. The independent predictive utility of computed tomography angiographic collateral status in acute ischaemic stroke. Brain. (2009) 132:2231–8. 10.1093/brain/awp155 19509116

[14] Tan BYQ Wan-Yee K Paliwal P Gopinathan A Nadarajah M Ting E. Good intracranial collaterals trump poor ASPECTS (Alberta stroke program early CT score) for intravenous thrombolysis in anterior circulation acute ischemic stroke. Stroke. (2016) 47:2292–8. 10.1161/STROKEAHA.116.013879 27491731

[15] Menon BK Smith EE Modi J Patel SK Bhatia R Watson TWJ. Regional leptomeningeal score on CT angiography predicts clinical and imaging outcomes in patients with acute anterior circulation occlusions. Am J Neuroradiol. (2011) 32:1640–5. 10.3174/ajnr.A2564 21799045 PMC7965388

[16] Cao R Qi P Liu Y Ma X Shen Z Chen J. Improving prognostic evaluation by 4D CTA for endovascular treatment in acute ischemic stroke patients: a preliminary study. J Stroke Cerebrovasc Dis. (2019) 28:1971–8. 10.1016/j.jstrokecerebrovasdis.2019.03.038 30981581

[17] Berkhemer OA Fransen PS Beumer D van den Berg LA Lingsma HF Yoo AJ. A randomized trial of intraarterial treatment for acute ischemic stroke. N Engl J Med. (2015) 372:11–20. 10.1056/NEJMoa1411587 25517348

[18] Yoo AJ Chaudhry ZA Nogueira RG Lev MH Schaefer PW Schwamm LH. Infarct volume is a pivotal biomarker after intra-arterial stroke therapy. Stroke. (2012) 43:1323–30. 10.1161/STROKEAHA.111.639401 22426317

[19] Rava RA Snyder KV Mokin M Waqas M Allman AB Senko JL. Assessment of a bayesian vitrea CT perfusion analysis to predict final infarct and penumbra volumes in patients with acute ischemic stroke: a comparison with RAPID. Am J Neuroradiol. (2020) 41:206–12. 10.3174/ajnr.A6395 31948951 PMC7015204

[20] Kaschka IN Kloska SP Struffert T Engelhorn T Gölitz P Kurka N. Clot burden and collaterals in anterior circulation stroke: differences between single-phase CTA and multi-phase 4D-CTA. Clin Neuroradiol. (2016) 26:309–15. 10.1007/s00062-014-0359-6 25410583

[21] Berkhemer OA Jansen IGH Beumer D Fransen PSS van den Berg LA Yoo AJ. Collateral status on baseline computed tomographic angiography and Intra-Arterial treatment effect in patients with proximal anterior circulation stroke. Stroke. (2016) 47:768–76. 10.1161/STROKEAHA.115.011788 26903582

[22] Bang OY Goyal M Liebeskind DS. Collateral circulation in ischemic stroke. Stroke. (2015) 46:3302–9. 10.1161/STROKEAHA.115.010508 26451027 PMC4624512

[23] Shi F Gong X Liu C Zeng Q Zhang M Chen Z. Acute stroke: prognostic value of quantitative collateral assessment at perfusion CT. Radiology. (2019) 290:760–8. 10.1148/radiol.2019181510 30620255

[24] Boers AMM Sales Barros R Jansen IGH Berkhemer OA Beenen LFM Menon BK. Value of quantitative collateral scoring on CT angiography in patients with acute ischemic stroke. Am J Neuroradiol. (2018) 39:1074–82. 10.3174/ajnr.A5623 29674417 PMC7410629

[25] Christoforidis GA Vakil P Ansari SA Dehkordi FH Carroll TJ. Impact of pial collaterals on infarct growth rate in experimental acute ischemic stroke. Am J Neuroradiol. (2017) 38:270–5. 10.3174/ajnr.A5003 27856435 PMC5826586

[26] Elijovich L Goyal N Mainali S Hoit D Arthur AS Whitehead M. CTA collateral score predicts infarct volume and clinical outcome after endovascular therapy for acute ischemic stroke: a retrospective chart review. J Neurointerv Surg. (2016) 8:559–62. 10.1136/neurintsurg-2015-011731 25994937

[27] Park H Kim BM Baek J Kim J Heo JH Kim DJ. Predictors of good outcomes in patients with failed endovascular thrombectomy. Korean J Radiol. (2020) 21:582. 10.3348/kjr.2019.0578 32323503 PMC7183835

[28] Kudo K Sasaki M Yamada K Momoshima S Utsunomiya H Shirato H. Differences in CT perfusion maps generated by different commercial software: Quantitative analysis by using identical source data of acute stroke patients. Radiology. (2010) 254:200–9. 10.1148/radiol.254082000 20032153

[29] Bivard A Levi C Spratt N Parsons M. Perfusion CT in acute stroke: a comprehensive analysis of infarct and penumbra. Radiology. (2013) 267:543–50. 10.1148/radiol.12120971. 10.1148/radiol.12120971 23264345

[30] Nogueira RG Jadhav AP Haussen DC Haussen DC Bonafe A Budzik RF. Thrombectomy 6 to 24 hours after stroke with a mismatch between deficit and infarct. N Engl J Med. (2018) 378:11–21 10.1056/NEJMoa1706442 29129157

[31] Ma H Campbell B Parsons MW Churilov L Levi CR Hsu Chung. Thrombolysis guided by perfusion imaging up to 9 hours after onset of stroke. N Engl J Med. (2019) 380:1795–803 10.1056/NEJMoa1813046 31067369

[32] Audebert HJ Sobesky J. Stroke: time is brain after stroke, regardless of age and severity. Nat Rev Neurol. (2014) 10:675–6. 10.1038/nrneurol.2014.194 25330727

[33] Munuera J Blasco G Hernández-Pérez M Daunis-I-Estadella P Dávalos A Liebeskind DS. Venous imaging-based biomarkers in acute ischemic stroke. J Neurol Neurosurg Psychiatry. (2016) 88:62–9. 10.1136/jnnp-2016-314814 27807197

